# Type-II diabetes and pancreatic cancer: a meta-analysis of 36 studies

**DOI:** 10.1038/sj.bjc.6602619

**Published:** 2005-05-10

**Authors:** R Huxley, A Ansary-Moghaddam, A Berrington de González, F Barzi, M Woodward

**Affiliations:** 1 The George Institute for International Health, The University of Sydney, PO Box M201, Missenden Road, Sydney NSW 2050, Australia; 2 Cancer Research UK Epidemiology Unit, University of Oxford, Gibson Building, Radcliffe Infirmary, Oxford OX2 6HE, UK

**Keywords:** pancreatic cancer, type-II diabetes, meta-analysis

## Abstract

Pancreatic cancer is the eighth major form of cancer-related death worldwide, causing 227 000 deaths annually. Type-II diabetes is widely considered to be associated with pancreatic cancer, but whether this represents a causal or consequential association is unclear. We conducted a meta-analysis to examine this association. A computer-based literature search from 1966 to 2005 yielded 17 case–control and 19 cohort or nested case–control studies with information on 9220 individuals with pancreatic cancer. The age and sex-adjusted odds ratio (OR) for pancreatic cancer associated with type-II diabetes was obtained from each study. The combined summary odds ratio was 1.82 (95% confidence interval (95% CI) 1.66–1.89), with evidence of heterogeneity across the studies (*P*=0.002 for case–control and *P*=0.05 for cohort studies) that was explained, in part, by higher risks being reported by smaller studies and studies that reported before 2000. Individuals in whom diabetes had only recently been diagnosed (<4 years) had a 50% greater risk of the malignancy compared with individuals who had diabetes for ⩾5 years (OR 2.1 *vs* 1.5; *P*=0.005). These results support a modest causal association between type-II diabetes and pancreatic cancer.

Although cancer of the pancreas accounts for only 3% of all cancers worldwide (Parkin *et al*, 2002), its poor prognosis makes it the eighth major form of cancer-related death worldwide, causing more than 220 000 deaths annually. Apart from cigarette smoking, which has been estimated to cause about 30% of pancreatic cancers (Doll and Peto, 1976; Baghurst *et al*, 1991), relatively little is known about the other chief determinants of the disease, although other lifestyle factors have been implicated, including obesity (Berrington de Gonzalez *et al*, 2003) and type-II diabetes (Everhart and Wright, 1995).

As type-II diabetes frequently occurs with pancreatic cancer, it is considered by many to be an important risk factor for the malignancy. A meta-analysis of 20 studies conducted a decade ago estimated that individuals with diabetes have a two-fold greater relative risk (RR) of pancreatic cancer compared with individuals without diabetes (Everhart and Wright, 1995). However, there is also good evidence that pancreatic cancer is causally linked to the onset of diabetes (Wang *et al*, 2003) and is possibly associated with an abnormality of islet cell function.

A large number of new studies have since published on the association between type-II diabetes and pancreatic cancer. Therefore, the purpose of the current study was to update the earlier meta-analysis to provide the most reliable information on the size of the association, and to explore any underlying sources of heterogeneity, including variations in the strength of the relationship with duration of diabetes.

## MATERIALS AND METHODS

### Data sources

Relevant studies were identified through EMBASE, PUBMED and MEDLINE using a combined text word and MESH heading search strategy of pancreatic cancer (pancreas, tumour, malignancy) and type-II diabetes (NIDDM, diabetes, adult-onset diabetes). References from identified studies, as well as from the previous review, were also scanned to identify any other relevant studies.

### Study selection and data synthesis

Studies were included in this systematic review if they had published quantitative estimates and standard errors, or confidence limits, of the association between type-II diabetes and pancreatic cancer by January 2005. Studies were excluded if they provided only an estimate of effect, with no means by which to calculate the standard error, or if the estimates were not adjusted by age. The variance of the log odds ratio (OR) from each study was calculated by converting the 95% confidence interval (CI) to its natural logarithm by taking the width of the CI and dividing by 3.92. If the variance was unavailable, *P*-values were used to estimate the CI. Some studies provided more than one OR according to the duration of diabetes before being diagnosed with pancreatic cancer. To maintain consistency across studies, the ORs for individuals diagnosed with diabetes >1 year prior to the diagnosis of pancreatic cancer were extracted and combined. Summary estimates were obtained separately for case–control and cohort studies by means of a ‘random effects’ approach and studies were weighted according to an estimate of its ‘statistical size’ defined as the inverse of the variance of the log OR (Woodward, 2005). In addition, studies that reported separate ORs for mutually exclusive categories of duration since diabetes was diagnosed (e.g. 1–4 years, 5–9 years, >10 years) were pooled separately to examine how the strength of the association varied with duration of diabetes. Possible sources of heterogeneity were investigated by comparing the results for studies combined with respect to particular characteristics (e.g. sex, method of diagnosis of diabetes). All analyses were performed using STATA, version 8.

## RESULTS

A total of 17 case–control and 19 cohort or nested case–control studies with information on a total of 9220 individuals with pancreatic cancer had published estimates of the association between diabetes and the malignancy. Six additional studies were excluded from this review; one study had published the standardised mortality ratio only (Kessler, 1970), and the remaining five studies contained duplicate information (La Vecchia *et al*, 1990; Davey Smith *et al*, 1992; Calle *et al*, 1998; Silverman *et al*, 1999; La Coughlin *et al*, 2000). The summary characteristics of included studies are shown in Tables 1 and 2. The majority of the study populations were from either North America (*n*=16) or Europe (*n*=14) with the remaining five studies from either Australasia or South America. With the exception of one study that had recorded diagnosis of diabetes by proxy, diagnosis of diabetes was either through self-report, an oral glucose-tolerance test, medical records or a combination of these three methods.

**Figure 1:**
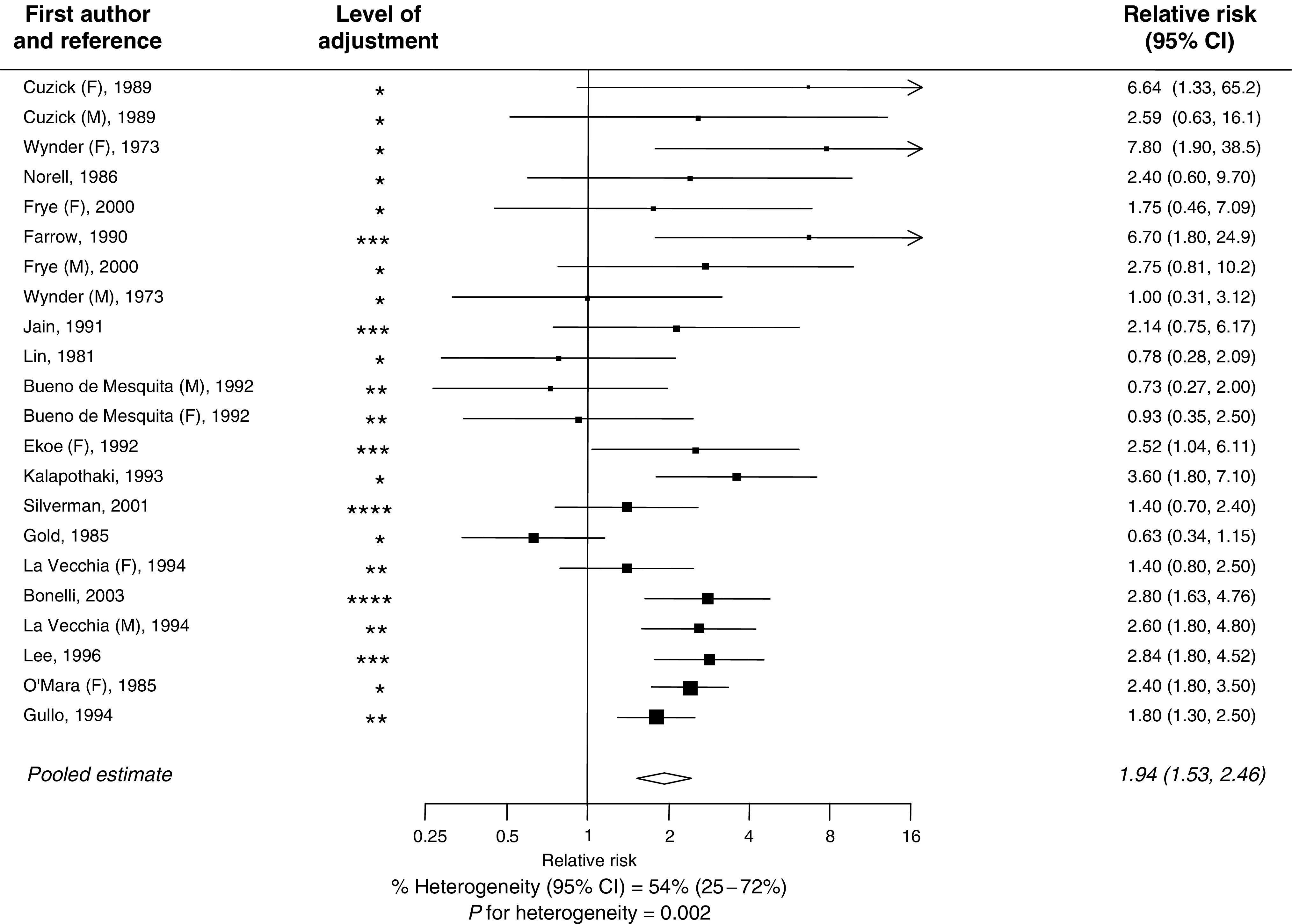
Relationship between type-II diabetes and risk of pancreatic cancer in case–control studies. Black square=point estimate (with area proportional to statistical ‘information’, based on inverse of variance of the OR provided by each study) and horizontal line=95% CI for observed effect in each study. ^*^=adjustment for age and sex; ^**^=adjustment for age, sex, smoking or a marker of social class; ^***^=adjustment for age, sex, smoking and social class; ^****^=adjustment for age, sex, smoking, social class and dietary variables.

**Figure 2:**
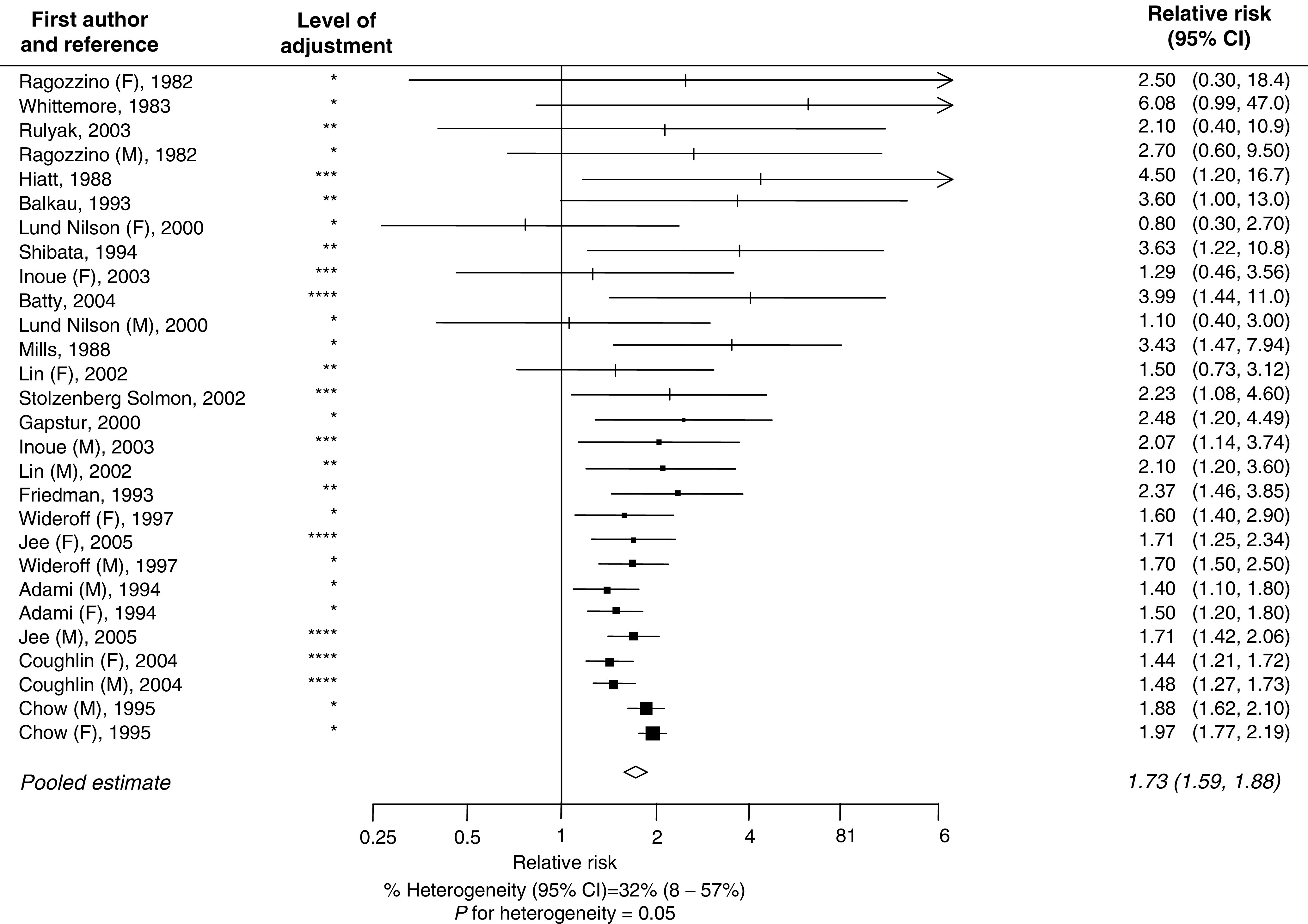
Relationship between type-II diabetes and risk of pancreatic cancer in cohort studies (conventions as in Figure 1).

**Figure 3:**
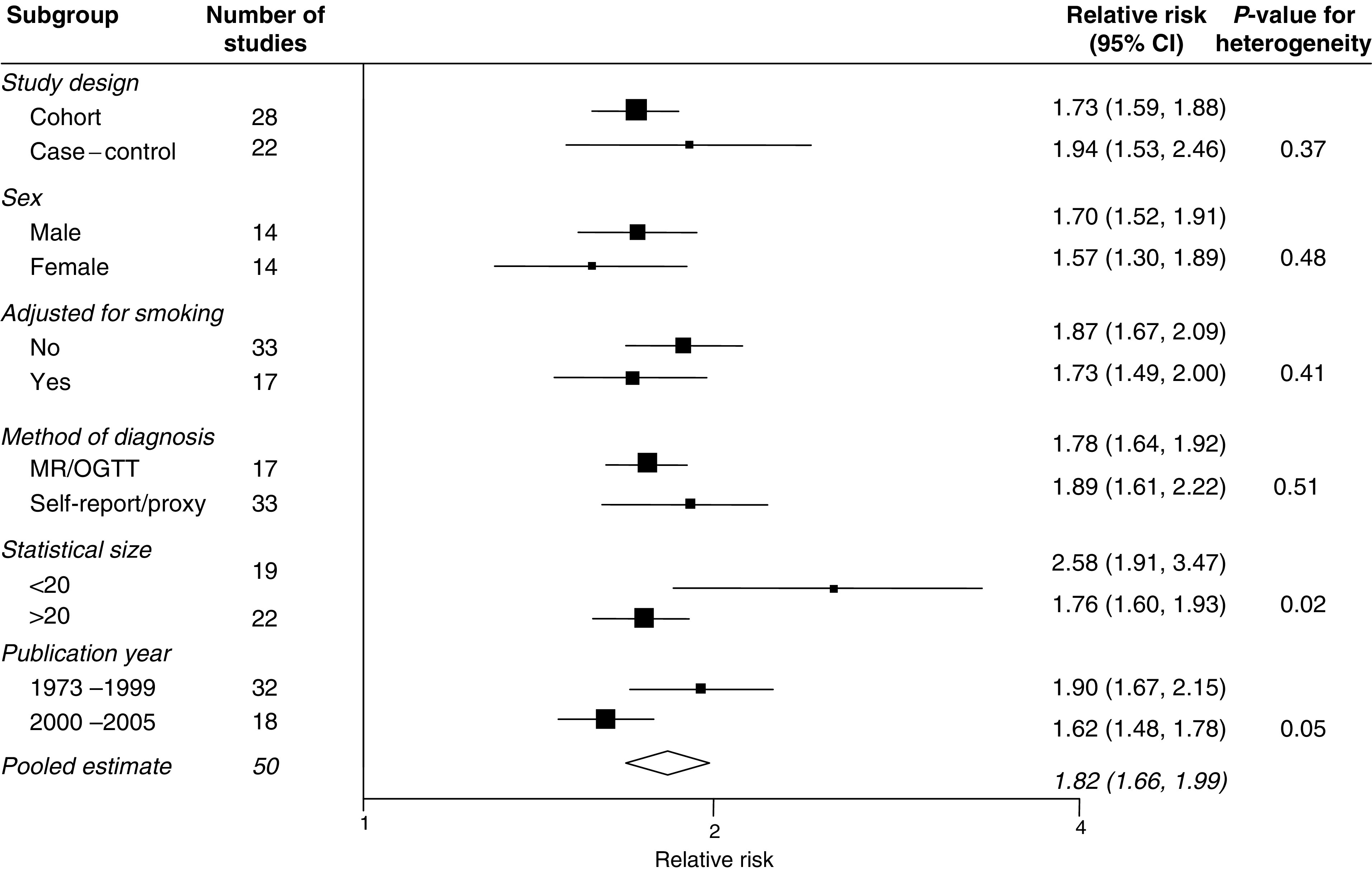
Sensitivity analyses (conventions as in Figure 1). MR=medical record; OGTT=oral glucose tolerance test; self-report=self-reported diabetes; proxy=diabetes status given by proxy.

The pooled OR for case–control studies was 1.94 (95% CI 1.53–2.46) (Figure 1), this being nonsignificantly higher than the summary estimate from cohort studies: 1.73 (1.59–1.88; Figure 2) (*P* for heterogeneity=0.37). The combined estimate from all studies was 1.82 (95% CI 1.66–1.99). There was some evidence of heterogeneity within both the case–control (*P*=0.002) and the cohort studies (*P*=0.05) that was that was not explained by differences in strength of effect between men and women, adjustment for cigarette smoking or the method of diagnosis of diabetes (Figure 3). Furthermore, there was no difference in the summary RR from those studies that had adjusted for variables other than age and sex compared to those studies that were unable to adjust for other potential confounders (RR 1.85 *vs* 1.80; *P*=0.78). There was some evidence to suggest that smaller studies tended to report higher RRs compared with larger studies (*P*=0.02). In addition, the summary estimate from studies that were published before 2000 was significantly higher than those studies published after this date: 1.90 (95% CI 1.67–2.15) *vs* 1.62 (95% CI 1.48–1.78): *P*=0.046.

**Figure 4:**
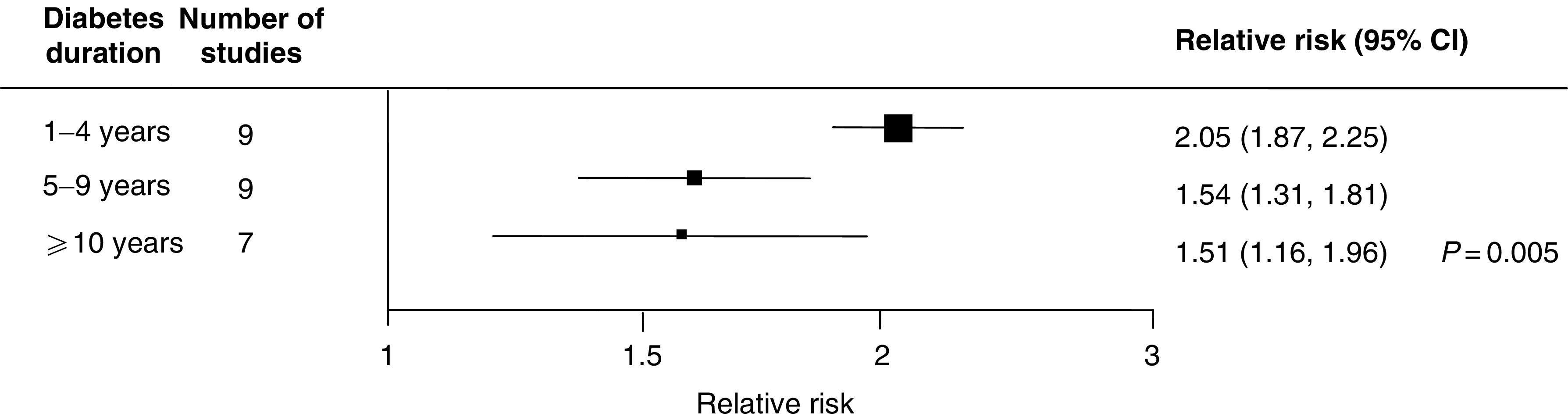
Relative risk of pancreatic cancer by duration of diabetes (conventions as in Figure 1).

Nine studies had reported mutually exclusive categories for the duration of diabetes that were broadly similar across the studies: <4 years, 5–9 years and >10 years. Individuals who had the shortest history of diabetes (<4 years) had more than a 50% greater risk of developing pancreatic cancer than individuals who had diabetes for between 5 and 10 years or for more than 10 years (OR 2.1 95% CI 1.9–2.3 *vs* OR 1.5 95% CI 1.3–1.8; Figure 4).

## DISCUSSION

The findings from this review suggest that earlier reports of a more than two-fold excess risk of pancreatic cancer among individuals with type-II diabetes are likely to have overestimated the strength of the association (Ragozzino *et al*, 1982; Friedman and Van Den Eeden, 1993; Everhart and Wright, 1995). Although the current data suggest an 80% greater risk of pancreatic cancer among individuals with type-II diabetes, even this may be an exaggeration of the true strength of the relationship, as it does not consider the considerable potential for reverse causality.

The RR of pancreatic cancer was demonstrated to be negatively associated with the duration of diabetes. Among individuals with a long history of diabetes (>5 years), the excess RR of pancreatic cancer was about 50% lower than in individuals for whom the duration of diabetes was shorter (RR 1.5 *vs* 2.1; *P*=0.005). This supports the hypothesis that, in some cases, diabetes may be an early manifestation of the tumour, as otherwise the RR would be expected to increase, rather than decrease, with duration of diabetes. In an earlier review similar RRs of pancreatic cancer by duration of diabetes were reported (Everhart and Wright, 1995) but, the categories for duration of diabetes, unlike in the current meta-analysis, were not mutually exclusive (i.e.>5 years diabetes duration was a subset of the >1 year group) and therefore, the RR for the >1 year duration of diabetes was likely to have been diluted by the inclusion of cases with a longer history of diabetes (and hence a smaller RR).

Evidence from the literature further supports the hypothesis that pancreatic cancer can induce a diabetic state. First, several studies have shown that the risk of pancreatic cancer among individuals with diabetes is lessened after exclusion of those with less that 1 year of diabetes, indicating that a component of the RR associated with diabetes is explained by individuals who are diagnosed with the malignancy within a short period of being diagnosed with diabetes (Ragozzino *et al*, 1982; Cuzick and Babiker, 1989). Second, in studies among individuals with pancreatic cancer who underwent tumour resection, insulin sensitivity and diabetes status were reported as showing substantial improvement 3 months after surgery (Permert *et al*, 1993). And third, molecular studies of sera from pancreatic cancer patients have identified peptides that are suggested to be diabetogenic (Wang *et al*, 2003).

Although the above arguments support the idea of reverse causality, the finding of a 50% increased RR of pancreatic cancer among individuals with chronic diabetes (>5 years) supports a modest causal relationship between diabetes and pancreatic cancer. This is true especially when considering that in individuals with a long history of diabetes (>5 years), it is unlikely that a malignancy that has a particularly low 1-year survival rate (fewer than 20% of individuals are alive at 1-year following diagnosis) could induce diabetes many years prior to its diagnosis. In addition, the presence of a graded dose–response association between fasting glucose and pancreatic cancer, reported by some large prospective studies, supports a causal relationship (Batty *et al*, 2004; Jee *et al*, 2005).

Inherent in any review process of published studies is the possibility of publication bias that may have resulted in an overestimate of the size of any association between two variables. There was some evidence to suggest that there was some publication bias such that the smaller studies were more likely to report a positive association between diabetes and pancreatic cancer. However, the exclusion of any small negative studies is unlikely to have materially altered the overall summary estimate. Confounding is also likely to have been present since diabetes and pancreatic cancer share several risk factors such as cigarette smoking and obesity. However, adjustment for a wide range of potential confounders only marginally attenuated the relationship between diabetes and pancreatic cancer. It is also possible that the use of aspirin and other nonsteroidal anti-inflammatory drugs (NSAIDS) may have been a potential confounder. For example, in a prospective follow-up study of over 28 000 postmenopausal women, women who reported using aspirin and other NSAIDS had a significantly lower RR of pancreatic cancer compared to non-users (RR 0.57 95% CI 0.36–0.90) (Anderson *et al*, 2002). In recent years, individuals with diabetes have been more likely to receive advice to take aspirin than people without diabetes in order to lower the risk of cardiovascular disease (CVD). For example, in the UK Prospective Diabetes Study, it was reported that between 1996/1997 and 2000/2001, aspirin use in patients without pre-existing CVD increased from 17 to 31% (*P*< 0.0001) (Cull *et al*, 2004). Aspirin has been reported to reduce the risk of several types of cancer (Garcia Rodriguez and Huerta-Alvarez, 1998; Thun *et al*, 1993); hence, the association between diabetes and pancreatic cancer may have been attenuated by aspirin use and indeed in studies published before 1995, the RR was significantly higher than in those published after this date (RR 2.3 *vs* 1.8).

Furthermore, most studies included in this review did not differentiate between Type-I and Type-II diabetes, which may have slightly underestimated the overall association, since it has been reported that Type-1 diabetes is not associated with pancreatic cancer (Zendehdel *et al*, 2003). The literature, however, regarding cancer mortality among individuals with type 1 diabetes, is limited by small sample size and short length of follow-up (Mihara *et al*, 1986; Martinenghi *et al*, 1997) and therefore do not preclude a possible association. However, it is likely that the substantial majority of individuals with diabetes included in these studies had type-II diabetes, since this is by far the most common form particularly in older individuals. Additional limitations of this review include the reliance, in the large majority of studies, on self-reported diabetes and the potential for misclassification on death certificates of site-specific cancers, although the sensitivity analyses did not show any difference in the risk between those studies that used self-reported diabetes compared with those that diagnosed diabetes either through medical records or by an oral glucose tolerance test.

To date, only cigarette smoking, and possibly obesity, has been identified as being causally associated with pancreatic cancer. The evidence from this review indicates that type-II diabetes is likely to be a third modifiable risk factor (Knowler *et al*, 2002; Davey Smith *et al*, 2005) and unless the increasing worldwide prevalence of all three risk factors is halted, the incidence of pancreatic cancer will rise substantially within the next couple of decades.

## Figures and Tables

**Table 1:** Case–control studies of diabetes and pancreatic cancer

	**PC cases**		**Controls**		**Diabetes**					
**First author and year**	**No.**	**Source**	**No.**	**Source**	**Source diagnosis**	**Duration (years)**	**% PC cases**	**Level of adjustment**	**Relative risk**	**95% CI**
Cuzick, 1989	216	Newly diagnosed patients, England	279	Hospital patients	SR, MR	>1	6.0 M	Age, sex	2.59	0.63–16.1
							2.0 F		6.64	1.33–65.2
Wynder, 1973	142	Patients, US	142	Hospital patients	SR	>2	5.0 M	Age, sex, race, hospital	1.00	0.31–3.12
							16.7 F		7.80	1.90–38.5
Norell, 1986	99	Newly diagnosed patients, Sweden	301	Community	SR	>5	4.0	Age, sex, residence	2.40	0.60–9.70
Frye, 2000	116	Patients, New Zealand	232	Hospital patients	MR	>1		Age, sex	2.75	0.81–10.2
									1.75	0.46–7.09
Farrow, 1990	148	Patients in cancer registry US	188	Community	P	>3	7.1	Age, smoking, education	6.70	1.80–24.9
Jain, 1991	249	Newly diagnosed patients, Canada	505	Community	SR, P	5–10	3.8	Age, sex, smoking, energy and fiber intake	2.14	0.75–6.17
Lin, 1981	109	Hospitalised patients, US	109	Hospital patients	SR	>1	7.4	Age, sex, race, marital status, hospital	0.78	0.28–2.09
Bueno de Mesquita, 1992	176	Patients, Netherlands	487	Community	SR, P	>1	6.4 M	Age, proxy, smoking	0.73	0.27–2.00
							7.3 F		0.93	0.35–2.50
Ekoe, 1992	179	Patients, Quebec	239	Community	SR	>1	NA	Age, sex, smoking, education	2.52	1.04–6.11
Kalapothaki, 1993	181	Hospitalised patients, Greece	362	Hospitalised patients	SR	>10	5.0	Age, sex, hospital	3.60	1.80–7.10
Silverman, 2001	526	Hospitalised patients, US	2153	Community	SR, P	2–4	NA	Age, sex, race, area, smoking, alcohol, BMI	1.40	0.70–2.40
Gold, 1985	201	Newly diagnosed patients, US	402	Hospitalised patients	SR, P	>2	10.0	Age, sex, race, hospital	0.63	0.34–1.15
La Vecchia, 1994	362	Hospitalised patients, Italy	1089	Hospitalised patients	SR	>1	14.4M	Age, sex, education, BMI	2.60	1.80–4.80
							9.8 F		1.40	0.80–2.50
Bonelli, 2003	202	Hospitalised patients, Italy	406	Hospitalised patients	SR	>1	18.3	Age, sex, centre, education, occupation, tobacco, alcohol	2.80	1.63–4.76
Lee, 1996	282	Hospitalised patients, Taipei	282	Hospital	SR	>1	28.7	Age, sex, smoking, alcohol	2.84	1.80–4.52
Gullo, 1994	720	Hospitalised patients, Italy	720	Hospitalised patients	SR, MR	>1	13.6	Age, social class, region, hospital	1.80	1.30–2.50
O'Mara, 1985	93	Hospitalised patients, US	NA	Hospitalised patients	SR	NA	7.6	Age	2.40	1.80–3.50

PC=pancreatic cancer; SR=self-reported diabetes; MR=medical record of diabetes; P=proxy provided information on diabetes status; NA=data not available.

**Table 2:** Cohort and nested case–control studies of diabetes and pancreatic cancer

		**Diabetes**			**PC among individuals with diabetes**				
**First author and year**	**Cohort source**	**Source of diagnosis**	**Duration**	**No. PY follow-up**	**Source**	**No. of cases**	**Level of adjustment**	**Relative risk**	**95% CI**
Ragozzino, 1982	Rochester, US	MR	>1	9800	MR, DC	3 M	Age, sex	2.70	0.60–9.50
						2 F		2.50	0.30–18.4
Whittemore, 1983	50 000 students, Harvard University, US	SR	>6	NA	DC	3	Age	6.08	0.99–47.0
Rulyak, 2003	251 subjects, Washington, US	SR	NA	NA	MR, P, DC	NA	Age, smoking, sex, prior history of non-PC	2.10	0.40–10.9
Hiatt, 1988	122 894, San Francisco, US	SR	>5	NA	MR, CR	5	Age, sex, race, smoking, alcohol, coffee	4.50	1.20–16.7
Balkau, 1993	6988 male civil servants, France	OGTT	>2	NA	MR, P	NA	Age, smoking	3.60	1.00–13.0
Shibata, 1994	13 976 Southern California retirees, US	SR	=<4	3057	MR	4	Age, sex, smoking	3.63	1.22–10.8
Inoue, 2003	200, Japan	SR	>1	NA	MR, CR	NA	Age, sex, family history, alcohol, exercise, diet	2.07	1.14–3.74
								1.29	0.46–3.56
Batty, 2004	18 006 male civil servants, UK	OGTT	NA	NA	DC	4	Age, employment, smoking, SBP, physical activity, disease history	3.99	1.44–11.0
Jee, 2005	1 298 385, Korea	OGTT	>1	NA	MR, CR	NA	Age, smoking, alcohol use	1.71	1.42–2.06
								1.71	1.25–2.34
Lund Nilsen 2000	31 000 M	SR	NA	6181	CR	4 M	Age, sex	1.10	0.40–3.00
	32 374 F, Norway			8059		3 F		0.80	0.30–2.70
Mills, 1988	34 000 Adventists, California	SR	>1	9683	DC	8	Age, sex	3.43	1.47–7.94
Gapstur, 2000	20 473 M, 15 183 F, Chicago	SR	NA	NA	DC	NA	Age	2.48	1.20–4.49
Stolzenberg-Solomon, 2002	29 084 M, Finland	SR	>5	10 669	CR, MR	14	Age, smoking, occupational activity, asthma, blood pressure	2.23	1.08–4.60
									
Lin, 2002	110 792, Japan	SR	>1	20 859 M, 15 389 F	DC	17	Age, sex, and smoking	2.10	1.20–3.60
						9		1.50	0.73–3.12
Friedman, 1993	175 000, San Francisco	SR	>1	NA	MR, CR		Age, weight	2.37	1.46–3.85
Adami, 1994	1.2 million, Sweden	Hospitalised patients	>1	143 618 M	CR	68 M	Age, sex	1.40	1.10–1.80
				119 643 F		88 F		1.50	1.20–1.80
Coughlin, 2004	467 922 M, 588 322 F, Columbia and Puerto Rico	SR	>1	NA	DC	NA	Age, sex, race, education, family history, BMI, smoking, alcohol, diet history	1.48	1.27–1.73
								1.44	1.21–1.72
Chow, 1995	134 096 hospitalised for diabetes, Sweden	Hospitalised patients	>1	432 643 M	CR	303	Age, sex, year of follow-up	1.88	1.62–2.10
				468493 F		347		1.97	1.77–2.19
Wideroff, 1997	109 581 hospitalised for diabetes, Denmark	Hospitalised patients	>1	628 129	MR, CR	417	Age, sex, year of follow-up	1.70	1.50–2.50
								1.60	1.40–2.90

PC=pancreatic cancer; MR=medical record of diabetes; SR=self-reported diabetes; OGTT=oral glucose tolerance test; P=proxy provided information on diabetes status; NA=data not available; DC=death certificate; CR=case-record form; M=male; F=female.
